# 4-Chloro-*N*-(2,5-dimethyl­phen­yl)benzene­sulfonamide

**DOI:** 10.1107/S1600536811019660

**Published:** 2011-05-28

**Authors:** K. Shakuntala, Sabine Foro, B. Thimme Gowda

**Affiliations:** a Department of Chemistry, Mangalore University, Mangalagangotri 574 199, Mangalore, India; b Institute of Materials Science, Darmstadt University of Technology, Petersenstrasse 23, D-64287 Darmstadt, Germany

## Abstract

The title compound, C_14_H_14_ClNO_2_S, contains two molecules in the asymmetric unit with different conformations. The C—SO_2_—NH—C torsion angles are 65.3 (2) and 54.6 (2)° and the aromatic rings are tilted relative to each other by 59.3 (1) and 45.8 (1)° in the two mol­ecules. In the crystal, inversion symmetry results in dimers linked by pairs of N—H⋯O hydrogen bonds for both molecules.

## Related literature

For hydrogen-bonding modes of sulfonamides, see: Adsmond & Grant (2001 ▶). For our studies of the effect of substituents upon the structures of *N*-(ar­yl)-amides, aryl­sulfonamides and methane­sulfonamides, see: Gowda *et al.* (2000 ▶, 2007 ▶, 2009 ▶); Shakuntala *et al.* (2011**a* ▶,b* ▶).
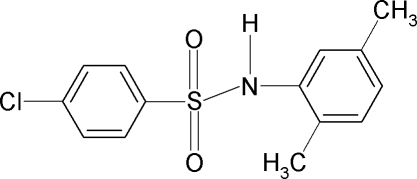


## Experimental

### 

#### Crystal data

C_14_H_14_ClNO_2_S
*M* *_r_* = 295.77
Triclinic, 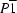

*a* = 10.624 (1) Å
*b* = 11.165 (1) Å
*c* = 13.845 (2) Å
α = 74.643 (8)°
β = 67.654 (7)°
γ = 82.195 (8)°
*V* = 1463.6 (3) Å^3^
*Z* = 4
Mo *K*α radiation
μ = 0.40 mm^−1^
*T* = 293 K
0.44 × 0.40 × 0.36 mm

#### Data collection

Oxford Diffraction Xcalibur diffractometer with a Sapphire CCD detector
Absorption correction: multi-scan (*CrysAlis RED*; Oxford Diffraction, 2009 ▶) *T*_min_ = 0.844, *T*_max_ = 0.869
10424 measured reflections
5971 independent reflections
4355 reflections with *I* > 2σ(*I*)
*R*_int_ = 0.012

#### Refinement

*R*[*F* ^2^ > 2σ(*F* ^2^)] = 0.040
*wR*(*F* ^2^) = 0.118
*S* = 1.03
5971 reflections
353 parameters
2 restraints
H atoms treated by a mixture of independent and constrained refinement
Δρ_max_ = 0.24 e Å^−3^
Δρ_min_ = −0.31 e Å^−3^

### 

Data collection: *CrysAlis CCD* (Oxford Diffraction, 2009 ▶); cell refinement: *CrysAlis RED* (Oxford Diffraction, 2009 ▶); data reduction: *CrysAlis RED*; program(s) used to solve structure: *SHELXS97* (Sheldrick, 2008 ▶); program(s) used to refine structure: *SHELXL97* (Sheldrick, 2008 ▶); molecular graphics: *PLATON* (Spek, 2009 ▶); software used to prepare material for publication: *SHELXL97*.

## Supplementary Material

Crystal structure: contains datablocks I, global. DOI: 10.1107/S1600536811019660/tk2748sup1.cif

Structure factors: contains datablocks I. DOI: 10.1107/S1600536811019660/tk2748Isup2.hkl

Supplementary material file. DOI: 10.1107/S1600536811019660/tk2748Isup3.cml

Additional supplementary materials:  crystallographic information; 3D view; checkCIF report

## Figures and Tables

**Table 1:** Hydrogen-bond geometry (Å, °)

*D*—H⋯*A*	*D*—H	H⋯*A*	*D*⋯*A*	*D*—H⋯*A*
N1—H1*N*⋯O2^i^	0.85 (2)	2.12 (2)	2.950 (2)	166 (2)
N2—H2*N*⋯O4^ii^	0.83 (2)	2.13 (2)	2.931 (2)	162 (2)

Symmetry codes: (i) 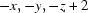; (ii) 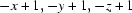.

**Fig. 1.**
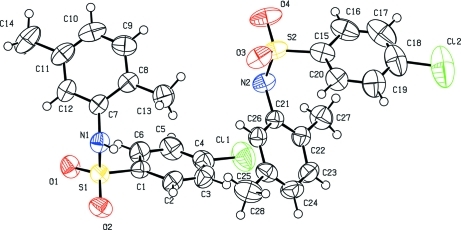
The molecular structure of (I), showing the atom-labelling scheme and displacement ellipsoids drawn at the 50% probability level.

**Fig. 2.**
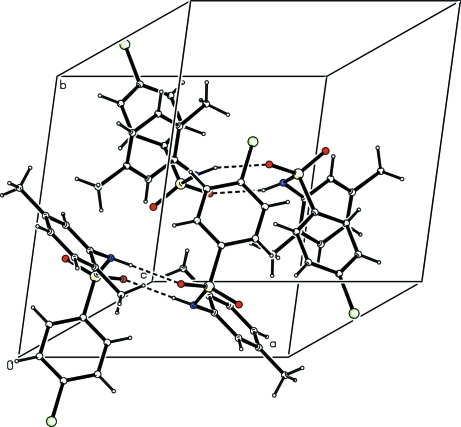
The molecular packing of (I) with hydrogen bonding shown as dashed lines.

## supplementary crystallographic information

### Comment

Sulfonamide moieties are constituents of many biologically important compounds. The hydrogen bonding preferences of sulfonamides have been investigated (Adsmond & Grant, 2001). As a part of our work on the substituent effects on the structures and other aspects of this class of compounds (Gowda *et al.*, 2000, 2007, 2009; Shakuntala *et al.*, 2011**a*,b*), in the present work, the crystal structure of 4-chloro-*N*-(2,5-dimethylphenyl)benzenesulfonamide (I) has been determined (Fig. 1). The asymmetric unit contains two independent molecules. In one of the molecules, the N—C bond in the C—SO_2_—NH—C segment has *gauche* torsions with respect to the S═O bonds. The molecules are twisted at the S atoms with the C—SO_2_—NH—C torsion angles of 65.3 (2) ° and 54.6 (2) ° in the two molecules, compared to the values of 34.7 (1) ° in 4-chloro-*N*-(2,3-dimethylphenyl)benzenesulfonamide (II) (Shakuntala *et al.*, 2011*b*), 69.1 (1) and 82.6 (1)° in the two independent molecules of 4-chloro-*N*-(phenyl)-benzenesulfonamide (III) (Shakuntala *et al.*, 2011*a*), and 62.7 (2) ° in *N*-(2,5-dimethylphenyl)benzenesulfonamide (IV) (Gowda *et al.*, 2009). Finally, the sulfonyl and the anilino benzene rings in the two independent molecules of (I) are tilted relative to each other by 59.3 (1) ° (molecule 1) and 45.8 (1) ° (molecule 2), compared to the values of -70.3 (3) ° in (II), -53.8 (3) ° and -63.4 (3) ° in the two independent molecules of (III), and 40.4 (1) ° in (IV).

In the crystal structure, inversion related molecules are linked by N—H···O hydrogen bonding into dimeric aggregates (Table 1 and Fig. 2).

### Experimental

A solution of chlorobenzene (10 ml) in chloroform (40 ml) was treated drop wise with chlorosulfonic acid (25 ml) at 273 K. After the initial evolution of hydrogen chloride subsided, the reaction mixture was brought to room temperature and poured into crushed ice in a beaker. The chloroform layer was separated, washed with cold water and allowed to evaporate slowly. The residual 4-chlorobenzenesulfonylchloride was treated with a stoichiometric amount of 2,5-dimethylaniline and boiled for 10 minutes. The reaction mixture was then cooled to room temperature and added to ice-cold water (100 ml). The resultant 4-chloro-*N*-(2,5-dimethylphenyl)benzenesulfonamide (I) was filtered under suction and washed thoroughly with cold water. It was then recrystallized to constant melting point from aqueous ethanol. Light-brown prisms of (I) were grown from its ethanolic solution by slow evaporation at room temperature.

### Refinement

The NH H-atoms were located in a difference map and were refined with the N—H distance restrained to 0.86 (2) Å. The other H atoms were positioned with idealized geometry using a riding model with the aromatic C—H = 0.93 Å and the methyl C—H = 0.96 Å. All H atoms were refined with isotropic displacement parameters set to 1.2*U*_eq_ of the parent atom.

### Crystal data

**Table:**

C_14_H_14_ClNO_2_S	*Z* = 4
*M**_r_* = 295.77	*F*(000) = 616
Triclinic, *P*1	*D*_x_ = 1.342 Mg m^−^^3^
Hall symbol: -P 1	Mo *K*α radiation, λ = 0.71073 Å
*a* = 10.624 (1) Å	Cell parameters from 4045 reflections
*b* = 11.165 (1) Å	θ = 2.9–27.8°
*c* = 13.845 (2) Å	µ = 0.40 mm^−^^1^
α = 74.643 (8)°	*T* = 293 K
β = 67.654 (7)°	Prism, light-brown
γ = 82.195 (8)°	0.44 × 0.40 × 0.36 mm
*V* = 1463.6 (3) Å^3^	

### Data collection

**Table:**

Oxford Diffraction Xcalibur diffractometer with a Sapphire CCD detector	5971 independent reflections
Radiation source: fine-focus sealed tube	4355 reflections with *I* > 2σ(*I*)
graphite	*R*_int_ = 0.012
Rotation method data acquisition using ω scans	θ_max_ = 26.4°, θ_min_ = 2.9°
Absorption correction: multi-scan (*CrysAlis RED*; Oxford Diffraction, 2009)	*h* = −13→13
*T*_min_ = 0.844, *T*_max_ = 0.869	*k* = −13→12
10424 measured reflections	*l* = −16→17

### Refinement

**Table:**

Refinement on *F*^2^	Primary atom site location: structure-invariant direct methods
Least-squares matrix: full	Secondary atom site location: difference Fourier map
*R*[*F*^2^ > 2σ(*F*^2^)] = 0.040	Hydrogen site location: inferred from neighbouring sites
*wR*(*F*^2^) = 0.118	H atoms treated by a mixture of independent and constrained refinement
*S* = 1.03	*w* = 1/[σ^2^(*F*_o_^2^) + (0.0645*P*)^2^ + 0.217*P*] where *P* = (*F*_o_^2^ + 2*F*_c_^2^)/3
5971 reflections	(Δ/σ)_max_ = 0.022
353 parameters	Δρ_max_ = 0.24 e Å^−^^3^
2 restraints	Δρ_min_ = −0.31 e Å^−^^3^

### Special details

**Table:**

Geometry. All e.s.d.'s (except the e.s.d. in the dihedral angle between two l.s. planes) are estimated using the full covariance matrix. The cell e.s.d.'s are taken into account individually in the estimation of e.s.d.'s in distances, angles and torsion angles; correlations between e.s.d.'s in cell parameters are only used when they are defined by crystal symmetry. An approximate (isotropic) treatment of cell e.s.d.'s is used for estimating e.s.d.'s involving l.s. planes.
Refinement. Refinement of *F*^2^ against ALL reflections. The weighted *R*-factor *wR* and goodness of fit *S* are based on *F*^2^, conventional *R*-factors *R* are based on *F*, with *F* set to zero for negative *F*^2^. The threshold expression of *F*^2^ > σ(*F*^2^) is used only for calculating *R*-factors(gt) *etc*. and is not relevant to the choice of reflections for refinement. *R*-factors based on *F*^2^ are statistically about twice as large as those based on *F*, and *R*- factors based on ALL data will be even larger.

### Fractional atomic coordinates and isotropic or equivalent isotropic displacement parameters (Å^2^)

**Table:**

	*x*	*y*	*z*	*U*_iso_*/*U*_eq_	
Cl1	0.36006 (9)	0.53177 (6)	0.87418 (6)	0.1008 (3)	
S1	0.20650 (4)	−0.02218 (5)	1.01469 (4)	0.04847 (14)	
O1	0.31155 (13)	−0.09429 (13)	1.04686 (11)	0.0597 (4)	
O2	0.06835 (12)	−0.03204 (15)	1.08715 (11)	0.0634 (4)	
N1	0.20717 (15)	−0.05945 (15)	0.90863 (14)	0.0505 (4)	
H1N	0.1347 (18)	−0.0308 (19)	0.8978 (17)	0.061*	
C1	0.24889 (17)	0.13475 (18)	0.97593 (14)	0.0455 (4)	
C2	0.1538 (2)	0.2278 (2)	0.96025 (17)	0.0558 (5)	
H2	0.0662	0.2077	0.9720	0.067*	
C3	0.1887 (2)	0.3495 (2)	0.92738 (18)	0.0635 (6)	
H3	0.1254	0.4126	0.9162	0.076*	
C4	0.3191 (2)	0.3775 (2)	0.91104 (17)	0.0617 (5)	
C5	0.4142 (2)	0.2861 (2)	0.92548 (19)	0.0697 (6)	
H5	0.5017	0.3066	0.9136	0.084*	
C6	0.3798 (2)	0.1642 (2)	0.95757 (18)	0.0619 (5)	
H6	0.4441	0.1014	0.9670	0.074*	
C7	0.32965 (17)	−0.06143 (17)	0.81716 (16)	0.0470 (4)	
C8	0.3468 (2)	0.02420 (19)	0.72070 (17)	0.0541 (5)	
C9	0.4666 (2)	0.0123 (2)	0.63506 (19)	0.0702 (6)	
H9	0.4815	0.0683	0.5691	0.084*	
C10	0.5638 (2)	−0.0801 (3)	0.6452 (2)	0.0747 (7)	
H10	0.6425	−0.0851	0.5860	0.090*	
C11	0.5473 (2)	−0.1649 (2)	0.7408 (2)	0.0668 (6)	
C12	0.42823 (19)	−0.15453 (18)	0.82663 (18)	0.0556 (5)	
H12	0.4139	−0.2112	0.8922	0.067*	
C13	0.2426 (3)	0.1260 (2)	0.7063 (2)	0.0774 (7)	
H13A	0.2250	0.1754	0.7576	0.093*	
H13B	0.1598	0.0900	0.7172	0.093*	
H13C	0.2766	0.1776	0.6350	0.093*	
C14	0.6526 (3)	−0.2670 (3)	0.7534 (3)	0.0968 (9)	
H14A	0.6897	−0.2547	0.8032	0.116*	
H14B	0.7242	−0.2652	0.6850	0.116*	
H14C	0.6108	−0.3460	0.7800	0.116*	
Cl2	0.09005 (14)	1.02785 (8)	0.31731 (10)	0.1497 (5)	
S2	0.29930 (4)	0.49101 (6)	0.46867 (4)	0.05870 (17)	
O3	0.20405 (14)	0.40575 (15)	0.48001 (13)	0.0677 (4)	
O4	0.43982 (14)	0.47354 (19)	0.40614 (14)	0.0838 (5)	
N2	0.29581 (15)	0.49000 (17)	0.58726 (14)	0.0545 (4)	
H2N	0.3663 (18)	0.517 (2)	0.5847 (18)	0.065*	
C15	0.2409 (2)	0.6403 (2)	0.41858 (16)	0.0589 (5)	
C16	0.3320 (3)	0.7294 (3)	0.34757 (19)	0.0790 (7)	
H16	0.4247	0.7086	0.3222	0.095*	
C17	0.2868 (4)	0.8466 (3)	0.3148 (2)	0.0987 (10)	
H17	0.3477	0.9064	0.2662	0.118*	
C18	0.1494 (4)	0.8763 (3)	0.3542 (2)	0.0922 (9)	
C19	0.0557 (3)	0.7888 (3)	0.4245 (2)	0.0815 (7)	
H19	−0.0368	0.8103	0.4499	0.098*	
C20	0.1023 (2)	0.6699 (2)	0.45579 (18)	0.0627 (6)	
H20	0.0412	0.6091	0.5019	0.075*	
C21	0.17227 (17)	0.52401 (17)	0.66661 (15)	0.0450 (4)	
C22	0.16013 (19)	0.63472 (18)	0.69746 (16)	0.0516 (5)	
C23	0.0385 (2)	0.6576 (2)	0.77699 (18)	0.0636 (6)	
H23	0.0280	0.7294	0.8018	0.076*	
C24	−0.0666 (2)	0.5780 (2)	0.82014 (18)	0.0641 (6)	
H24	−0.1468	0.5972	0.8729	0.077*	
C25	−0.05576 (19)	0.4699 (2)	0.78675 (16)	0.0578 (5)	
C26	0.06623 (18)	0.44264 (18)	0.71042 (15)	0.0502 (4)	
H26	0.0774	0.3690	0.6882	0.060*	
C27	0.2709 (2)	0.7278 (2)	0.6475 (2)	0.0720 (6)	
H27A	0.3544	0.6876	0.6533	0.086*	
H27B	0.2455	0.7941	0.6842	0.086*	
H27C	0.2834	0.7608	0.5731	0.086*	
C28	−0.1723 (2)	0.3822 (3)	0.8332 (2)	0.0877 (8)	
H28A	−0.2081	0.3685	0.9100	0.105*	
H28B	−0.1398	0.3044	0.8134	0.105*	
H28C	−0.2427	0.4180	0.8055	0.105*	

### Atomic displacement parameters (Å^2^)

**Table:**

	*U*^11^	*U*^22^	*U*^33^	*U*^12^	*U*^13^	*U*^23^
Cl1	0.1377 (7)	0.0670 (4)	0.1017 (5)	−0.0271 (4)	−0.0484 (5)	−0.0069 (4)
S1	0.0310 (2)	0.0595 (3)	0.0527 (3)	−0.00033 (18)	−0.0184 (2)	−0.0050 (2)
O1	0.0427 (7)	0.0663 (9)	0.0697 (9)	0.0027 (6)	−0.0308 (7)	−0.0015 (7)
O2	0.0353 (7)	0.0890 (11)	0.0576 (8)	−0.0060 (6)	−0.0148 (6)	−0.0044 (7)
N1	0.0350 (8)	0.0568 (10)	0.0635 (10)	0.0006 (7)	−0.0234 (7)	−0.0131 (8)
C1	0.0349 (9)	0.0585 (11)	0.0458 (10)	0.0029 (7)	−0.0177 (8)	−0.0144 (8)
C2	0.0466 (11)	0.0637 (13)	0.0664 (13)	0.0083 (9)	−0.0284 (10)	−0.0237 (10)
C3	0.0696 (14)	0.0636 (14)	0.0691 (14)	0.0143 (11)	−0.0379 (12)	−0.0241 (11)
C4	0.0773 (15)	0.0615 (13)	0.0491 (11)	−0.0110 (11)	−0.0230 (11)	−0.0128 (10)
C5	0.0529 (12)	0.0781 (16)	0.0766 (15)	−0.0165 (11)	−0.0232 (11)	−0.0090 (12)
C6	0.0396 (10)	0.0680 (14)	0.0762 (14)	0.0011 (9)	−0.0247 (10)	−0.0096 (11)
C7	0.0394 (9)	0.0480 (10)	0.0613 (12)	−0.0007 (7)	−0.0222 (9)	−0.0199 (9)
C8	0.0525 (11)	0.0582 (12)	0.0592 (12)	−0.0001 (9)	−0.0247 (10)	−0.0204 (10)
C9	0.0687 (15)	0.0865 (17)	0.0580 (13)	−0.0067 (12)	−0.0207 (11)	−0.0226 (12)
C10	0.0515 (13)	0.1006 (19)	0.0762 (16)	0.0014 (12)	−0.0118 (11)	−0.0465 (15)
C11	0.0479 (12)	0.0732 (15)	0.0932 (18)	0.0087 (10)	−0.0277 (12)	−0.0443 (14)
C12	0.0502 (11)	0.0478 (11)	0.0767 (14)	0.0029 (8)	−0.0288 (10)	−0.0208 (10)
C13	0.0839 (17)	0.0796 (17)	0.0654 (14)	0.0161 (13)	−0.0341 (13)	−0.0106 (12)
C14	0.0618 (15)	0.093 (2)	0.144 (3)	0.0291 (14)	−0.0373 (16)	−0.0580 (19)
Cl2	0.2453 (13)	0.0760 (5)	0.1754 (10)	−0.0184 (6)	−0.1439 (10)	0.0009 (6)
S2	0.0326 (2)	0.0867 (4)	0.0650 (3)	−0.0024 (2)	−0.0138 (2)	−0.0367 (3)
O3	0.0480 (8)	0.0808 (10)	0.0881 (11)	−0.0032 (7)	−0.0245 (7)	−0.0420 (9)
O4	0.0359 (8)	0.1438 (16)	0.0843 (11)	0.0026 (8)	−0.0118 (7)	−0.0648 (11)
N2	0.0333 (8)	0.0728 (11)	0.0646 (11)	0.0008 (7)	−0.0191 (8)	−0.0271 (9)
C15	0.0503 (11)	0.0830 (15)	0.0516 (11)	−0.0199 (10)	−0.0201 (9)	−0.0176 (10)
C16	0.0724 (15)	0.108 (2)	0.0584 (14)	−0.0385 (15)	−0.0176 (12)	−0.0143 (14)
C17	0.124 (3)	0.111 (3)	0.0714 (17)	−0.059 (2)	−0.0420 (18)	0.0003 (17)
C18	0.146 (3)	0.0692 (17)	0.0907 (19)	−0.0251 (17)	−0.079 (2)	−0.0017 (14)
C19	0.0865 (18)	0.0819 (18)	0.0928 (19)	−0.0031 (14)	−0.0538 (16)	−0.0153 (15)
C20	0.0518 (12)	0.0731 (15)	0.0692 (14)	−0.0102 (10)	−0.0297 (10)	−0.0105 (11)
C21	0.0376 (9)	0.0522 (11)	0.0472 (10)	0.0033 (7)	−0.0189 (8)	−0.0118 (8)
C22	0.0525 (11)	0.0521 (11)	0.0546 (11)	0.0000 (8)	−0.0248 (9)	−0.0122 (9)
C23	0.0683 (14)	0.0615 (13)	0.0644 (13)	0.0138 (11)	−0.0253 (11)	−0.0264 (11)
C24	0.0456 (11)	0.0851 (16)	0.0550 (12)	0.0095 (11)	−0.0122 (9)	−0.0203 (11)
C25	0.0438 (10)	0.0742 (14)	0.0512 (11)	−0.0052 (9)	−0.0175 (9)	−0.0059 (10)
C26	0.0462 (10)	0.0489 (11)	0.0570 (11)	−0.0004 (8)	−0.0222 (9)	−0.0102 (9)
C27	0.0792 (16)	0.0642 (14)	0.0781 (16)	−0.0187 (11)	−0.0282 (13)	−0.0175 (12)
C28	0.0626 (15)	0.111 (2)	0.0774 (17)	−0.0310 (14)	−0.0087 (13)	−0.0132 (15)

### Geometric parameters (Å, °)

**Table:**

Cl1—C4	1.728 (2)	Cl2—C18	1.737 (3)
S1—O1	1.4270 (13)	S2—O3	1.4200 (15)
S1—O2	1.4277 (13)	S2—O4	1.4296 (14)
S1—N1	1.6261 (17)	S2—N2	1.6252 (18)
S1—C1	1.759 (2)	S2—C15	1.755 (2)
N1—C7	1.432 (2)	N2—C21	1.436 (2)
N1—H1N	0.845 (15)	N2—H2N	0.830 (15)
C1—C2	1.382 (3)	C15—C16	1.383 (3)
C1—C6	1.385 (3)	C15—C20	1.387 (3)
C2—C3	1.368 (3)	C16—C17	1.353 (4)
C2—H2	0.9300	C16—H16	0.9300
C3—C4	1.382 (3)	C17—C18	1.377 (4)
C3—H3	0.9300	C17—H17	0.9300
C4—C5	1.367 (3)	C18—C19	1.385 (4)
C5—C6	1.369 (3)	C19—C20	1.370 (3)
C5—H5	0.9300	C19—H19	0.9300
C6—H6	0.9300	C20—H20	0.9300
C7—C8	1.384 (3)	C21—C22	1.386 (3)
C7—C12	1.390 (3)	C21—C26	1.389 (3)
C8—C9	1.390 (3)	C22—C23	1.388 (3)
C8—C13	1.504 (3)	C22—C27	1.509 (3)
C9—C10	1.376 (3)	C23—C24	1.369 (3)
C9—H9	0.9300	C23—H23	0.9300
C10—C11	1.373 (4)	C24—C25	1.379 (3)
C10—H10	0.9300	C24—H24	0.9300
C11—C12	1.384 (3)	C25—C26	1.382 (3)
C11—C14	1.509 (3)	C25—C28	1.516 (3)
C12—H12	0.9300	C26—H26	0.9300
C13—H13A	0.9600	C27—H27A	0.9600
C13—H13B	0.9600	C27—H27B	0.9600
C13—H13C	0.9600	C27—H27C	0.9600
C14—H14A	0.9600	C28—H28A	0.9600
C14—H14B	0.9600	C28—H28B	0.9600
C14—H14C	0.9600	C28—H28C	0.9600
			
O1—S1—O2	119.67 (9)	O3—S2—O4	119.34 (10)
O1—S1—N1	108.40 (9)	O3—S2—N2	108.59 (10)
O2—S1—N1	104.99 (8)	O4—S2—N2	105.32 (9)
O1—S1—C1	107.65 (8)	O3—S2—C15	107.35 (9)
O2—S1—C1	108.48 (9)	O4—S2—C15	109.38 (11)
N1—S1—C1	107.02 (9)	N2—S2—C15	106.13 (9)
C7—N1—S1	121.67 (12)	C21—N2—S2	120.23 (12)
C7—N1—H1N	118.1 (15)	C21—N2—H2N	115.1 (16)
S1—N1—H1N	109.0 (14)	S2—N2—H2N	112.1 (16)
C2—C1—C6	120.22 (19)	C16—C15—C20	120.4 (2)
C2—C1—S1	120.27 (14)	C16—C15—S2	120.56 (19)
C6—C1—S1	119.47 (15)	C20—C15—S2	118.96 (16)
C3—C2—C1	119.89 (19)	C17—C16—C15	120.2 (3)
C3—C2—H2	120.1	C17—C16—H16	119.9
C1—C2—H2	120.1	C15—C16—H16	119.9
C2—C3—C4	119.20 (19)	C16—C17—C18	119.2 (3)
C2—C3—H3	120.4	C16—C17—H17	120.4
C4—C3—H3	120.4	C18—C17—H17	120.4
C5—C4—C3	121.3 (2)	C17—C18—C19	121.9 (3)
C5—C4—Cl1	119.91 (18)	C17—C18—Cl2	119.9 (3)
C3—C4—Cl1	118.74 (18)	C19—C18—Cl2	118.3 (3)
C4—C5—C6	119.5 (2)	C20—C19—C18	118.5 (3)
C4—C5—H5	120.2	C20—C19—H19	120.8
C6—C5—H5	120.2	C18—C19—H19	120.8
C5—C6—C1	119.80 (19)	C19—C20—C15	119.8 (2)
C5—C6—H6	120.1	C19—C20—H20	120.1
C1—C6—H6	120.1	C15—C20—H20	120.1
C8—C7—C12	120.94 (19)	C22—C21—C26	121.60 (18)
C8—C7—N1	120.78 (17)	C22—C21—N2	120.70 (17)
C12—C7—N1	118.22 (18)	C26—C21—N2	117.70 (17)
C7—C8—C9	116.84 (19)	C21—C22—C23	116.47 (18)
C7—C8—C13	122.87 (19)	C21—C22—C27	122.79 (19)
C9—C8—C13	120.3 (2)	C23—C22—C27	120.73 (19)
C10—C9—C8	121.8 (2)	C24—C23—C22	122.1 (2)
C10—C9—H9	119.1	C24—C23—H23	118.9
C8—C9—H9	119.1	C22—C23—H23	118.9
C11—C10—C9	121.5 (2)	C23—C24—C25	121.2 (2)
C11—C10—H10	119.2	C23—C24—H24	119.4
C9—C10—H10	119.2	C25—C24—H24	119.4
C10—C11—C12	117.3 (2)	C24—C25—C26	117.83 (19)
C10—C11—C14	122.3 (2)	C24—C25—C28	121.5 (2)
C12—C11—C14	120.5 (3)	C26—C25—C28	120.7 (2)
C11—C12—C7	121.6 (2)	C25—C26—C21	120.71 (19)
C11—C12—H12	119.2	C25—C26—H26	119.6
C7—C12—H12	119.2	C21—C26—H26	119.6
C8—C13—H13A	109.5	C22—C27—H27A	109.5
C8—C13—H13B	109.5	C22—C27—H27B	109.5
H13A—C13—H13B	109.5	H27A—C27—H27B	109.5
C8—C13—H13C	109.5	C22—C27—H27C	109.5
H13A—C13—H13C	109.5	H27A—C27—H27C	109.5
H13B—C13—H13C	109.5	H27B—C27—H27C	109.5
C11—C14—H14A	109.5	C25—C28—H28A	109.5
C11—C14—H14B	109.5	C25—C28—H28B	109.5
H14A—C14—H14B	109.5	H28A—C28—H28B	109.5
C11—C14—H14C	109.5	C25—C28—H28C	109.5
H14A—C14—H14C	109.5	H28A—C28—H28C	109.5
H14B—C14—H14C	109.5	H28B—C28—H28C	109.5
			
O1—S1—N1—C7	−50.51 (17)	O3—S2—N2—C21	−60.53 (18)
O2—S1—N1—C7	−179.50 (15)	O4—S2—N2—C21	170.55 (16)
C1—S1—N1—C7	65.34 (16)	C15—S2—N2—C21	54.62 (17)
O1—S1—C1—C2	−167.76 (15)	O3—S2—C15—C16	−144.82 (18)
O2—S1—C1—C2	−36.91 (18)	O4—S2—C15—C16	−14.0 (2)
N1—S1—C1—C2	75.89 (17)	N2—S2—C15—C16	99.20 (19)
O1—S1—C1—C6	14.51 (19)	O3—S2—C15—C20	38.66 (19)
O2—S1—C1—C6	145.36 (16)	O4—S2—C15—C20	169.51 (16)
N1—S1—C1—C6	−101.84 (17)	N2—S2—C15—C20	−77.32 (18)
C6—C1—C2—C3	−0.6 (3)	C20—C15—C16—C17	0.8 (3)
S1—C1—C2—C3	−178.30 (16)	S2—C15—C16—C17	−175.64 (19)
C1—C2—C3—C4	−0.4 (3)	C15—C16—C17—C18	0.9 (4)
C2—C3—C4—C5	1.0 (3)	C16—C17—C18—C19	−1.7 (4)
C2—C3—C4—Cl1	−177.72 (16)	C16—C17—C18—Cl2	177.4 (2)
C3—C4—C5—C6	−0.5 (4)	C17—C18—C19—C20	0.6 (4)
Cl1—C4—C5—C6	178.20 (18)	Cl2—C18—C19—C20	−178.53 (18)
C4—C5—C6—C1	−0.6 (4)	C18—C19—C20—C15	1.2 (4)
C2—C1—C6—C5	1.1 (3)	C16—C15—C20—C19	−1.9 (3)
S1—C1—C6—C5	178.82 (17)	S2—C15—C20—C19	174.61 (18)
S1—N1—C7—C8	−112.12 (18)	S2—N2—C21—C22	−109.42 (18)
S1—N1—C7—C12	70.4 (2)	S2—N2—C21—C26	70.3 (2)
C12—C7—C8—C9	−0.1 (3)	C26—C21—C22—C23	2.2 (3)
N1—C7—C8—C9	−177.55 (17)	N2—C21—C22—C23	−178.08 (17)
C12—C7—C8—C13	179.6 (2)	C26—C21—C22—C27	−176.96 (19)
N1—C7—C8—C13	2.2 (3)	N2—C21—C22—C27	2.7 (3)
C7—C8—C9—C10	0.0 (3)	C21—C22—C23—C24	−2.5 (3)
C13—C8—C9—C10	−179.7 (2)	C27—C22—C23—C24	176.7 (2)
C8—C9—C10—C11	−0.2 (4)	C22—C23—C24—C25	0.5 (3)
C9—C10—C11—C12	0.5 (3)	C23—C24—C25—C26	1.8 (3)
C9—C10—C11—C14	179.9 (2)	C23—C24—C25—C28	−179.2 (2)
C10—C11—C12—C7	−0.6 (3)	C24—C25—C26—C21	−2.1 (3)
C14—C11—C12—C7	−180.0 (2)	C28—C25—C26—C21	179.0 (2)
C8—C7—C12—C11	0.4 (3)	C22—C21—C26—C25	0.1 (3)
N1—C7—C12—C11	177.91 (17)	N2—C21—C26—C25	−179.65 (17)

### Hydrogen-bond geometry (Å, °)

**Table:**

*D*—H···*A*	*D*—H	H···*A*	*D*···*A*	*D*—H···*A*
N1—H1N···O2^i^	0.85 (2)	2.12 (2)	2.950 (2)	166 (2)
N2—H2N···O4^ii^	0.83 (2)	2.13 (2)	2.931 (2)	162 (2)

Symmetry codes: (i) −*x*, −*y*, −*z*+2; (ii) −*x*+1, −*y*+1, −*z*+1.
